# Supplementary material for: Molecular basis of synaptic specificity by immunoglobulin superfamily receptors in Drosophila
Source: eLife. 2019 Jan 28;8:e41028. doi: 10.7554/eLife.41028 (PMC6374074; doi:10.7554/eLife.41028)
Supplement: Figure 7—source data 1. [file elife-41028-fig7-data1.docx]

**Figure 7–source data 2. Source data for Figure 7–figure supplement 7a, 7b and 7f.**

| Fig 7–fig. suppl. 1 | Genotype | Mean | Std. Dev. | S.E.M. | n (animals / hemisegments) | p-value |
| --- | --- | --- | --- | --- | --- | --- |
| a | DIP-α-GAL4> *DIP-α* | 100 | 18.74 | 5.197 | 6/13 | n/a* |
|  | DIP-α-GAL4> *DIP- α*^I83A^ | 118.1 | 43.55 | 12.57 | 6/12 | NS^†^ |
| b | UAS-*DIP-α* (female) | 81.36 | 39.28 | 5.114 | 6/59 | n/a |
|  | UAS-*DIP-α* (male) | 82.46 | 38.37 | 5.083 | 6/57 | NS |
|  | UAS-*DIP-α*^I83A^ (female) | 96.43 | 18.73 | 2.502 | 6/56 | n/a |
|  | UAS-*DIP-α*^I83A^ (male) | 87.27 | 33.63 | 4.535 | 6/55 | NS |
|  | *UAS-Dpr10* (female) | 91.67 | 27.87 | 3.598 | 6/60 | n/a |
|  | *UAS-Dpr10* (male) | 83.33 | 37.58 | 4.852 | 6/60 | NS |
|  | UAS-*Dpr10^Y103A^* (female) | 89.29 | 31.21 | 4.171 | 6/56 | n/a |
|  | UAS-*Dpr10^Y103A^* (male) | 83.53 | 37.31 | 4.047 | 9/85 | NS |
| f | *Eve-GAL4*>*DIP-α* | 93.1 | 25.45 | 2.363 | 12/116 | n/a |
|  | *Eve-GAL4*>*DIP-α*^I83A^ | 99.14 | 9.285 | 0.8621 | 12/116 | NS |

* not applicable

^†^ not significant
