# Supplementary material for: Molecular basis of synaptic specificity by immunoglobulin superfamily receptors in Drosophila
Source: eLife. 2019 Jan 28;8:e41028. doi: 10.7554/eLife.41028 (PMC6374074; doi:10.7554/eLife.41028)
Supplement: Figure 7—source data 2. [file elife-41028-fig7-data2.docx]

**Figure 7–source data 1. Source data for Figure 7g.**

| Fig. | Genotype | Mean | Std. Dev. | S.E.M. | n (animals / hemisegments) | p-value |
| --- | --- | --- | --- | --- | --- | --- |
| 7g | *DIP-α-GAL4>EGFP* (Het) | 74.07 | 44.23 | 6.02 | 7/54 | n/a* |
|  | *DIP-α-GAL4>EGFP* (Hemi) | 0 | 0 | 0 | 8/64 | <0.0001 |
|  | UAS-*DIP-α* | 81.9 | 38.67 | 3.591 | 12/116 | n/a |
|  | *DIP-α-GAL4> DIP-α* (Het) | 95.74 | 20.4 | 2.976 | 6/47 | NS^†^ |
|  | *DIP-α-GAL4> DIP-α* (Hemi) | 93.62 | 24.71 | 3.604 | 6/47 | NS |
|  | UAS-*DIP-**α*^I83A^ | 91.89 | 27.42 | 2.603 | 12/111 | n/a |
|  | *DIP-α-GAL4> DIP- α*^I83A^ (Het) | 87.27 | 33.63 | 4.535 | 7/55 | NS |
|  | *DIP-α-GAL4> DIP- α*^I83A^ (Hemi) | 0 | 0 | 0 | 7/56 | <0.0001 |

* not applicable

^†^ not significant
