# Supplementary material for: Molecular basis of synaptic specificity by immunoglobulin superfamily receptors in Drosophila
Source: eLife. 2019 Jan 28;8:e41028. doi: 10.7554/eLife.41028 (PMC6374074; doi:10.7554/eLife.41028)
Supplement: Figure 8—source data 1. [file elife-41028-fig8-data1.docx]

**Figure 8–source data 1. Source data for Figure 8c.**

| Fig. | Genotype | Mean | Std. Dev. | S.E.M. | n (animals / hemisegments) | p-value |
| --- | --- | --- | --- | --- | --- | --- |
| 8c | UAS*-dpr10* | 87.5 | 33.21 | 3.032 | 12/120 | n/a* |
|  | *Mef2-GAL4*>*dpr10* | 24.68 | 43.39 | 4.945 | 11/77 | <0.0001 |
|  | UAS-*dpr10^Y103A^* | 85.82 | 35.01 | 2.949 | 15/141 | n/a |
|  | *Mef2-GAL4>dpr10^Y103A^* | 88.68 | 31.99 | 4.394 | 7/53 | NS^†^ |
|  | *DIP-α^1-178^*/+; UAS-*dpr10* | 60.36 | 49.14 | 4.664 | 17/111 | n/a |
|  | *DIP-α^1-178^*/+; *Mef2-GAL4*>*dpr10* | 5.882 | 23.62 | 2.025 | 17/136 | <0.0001 |
|  | *DIP-α^1-178^*/+; UAS-*dpr10^Y103A^* | 61.67 | 48.82 | 4.457 | 1/120 | n/a |
|  | *DIP-α^1-178^*/+; *Mef2-GAL4*>*dpr10^Y103A^* | 46.43 | 50.32 | 6.725 | 7/56 | NS |

* not applicable

^†^ not significant
