# Supplementary material for: Molecular basis of synaptic specificity by immunoglobulin superfamily receptors in Drosophila
Source: eLife. 2019 Jan 28;8:e41028. doi: 10.7554/eLife.41028 (PMC6374074; doi:10.7554/eLife.41028)
Supplement: Figure 8—source data 2. [file elife-41028-fig8-data2.docx]

**Figure 8–source data 2. Source data for Figure 8–figure supplement 1.**

| Fig 8–fig. suppl. 1 | Genotype | Mean | Std. Dev. | S.E.M. | N (animals / hemisegments) | p-value |
| --- | --- | --- | --- | --- | --- | --- |
|  | BG487-*GAL4>dpr10* | 79.17 | 41.04 | 5.924 | 8/48 | n/a* |
|  | 24B-*GAL4>dpr10* | 15.63 | 36.89 | 6.521 | 8/32 | <0.0001 |

* not applicable
